# Routine development of objectively derived search strategies

**DOI:** 10.1186/2046-4053-1-19

**Published:** 2012-02-29

**Authors:** Elke Hausner, Siw Waffenschmidt, Thomas Kaiser, Michael Simon

**Affiliations:** 1 Institute for Quality and Efficiency in Health Care, Dillenburger Strasse 27, D-51105 Cologne, Germany; 2 University of Southampton, Faculty of Health Sciences, Southampton, SO17 1BJ, UK

**Keywords:** information storage and retrieval, reproducibility of results, bibliographic databases, health technology assessment

## Abstract

**Background:**

Over the past few years, information retrieval has become more and more professionalized, and information specialists are considered full members of a research team conducting systematic reviews. Research groups preparing systematic reviews and clinical practice guidelines have been the driving force in the development of search strategies, but open questions remain regarding the transparency of the development process and the available resources. An empirically guided approach to the development of a search strategy provides a way to increase transparency and efficiency.

**Methods:**

Our aim in this paper is to describe the empirically guided development process for search strategies as applied by the German Institute for Quality and Efficiency in Health Care (Institut für Qualität und Wirtschaftlichkeit im Gesundheitswesen, or "IQWiG"). This strategy consists of the following steps: generation of a test set, as well as the development, validation and standardized documentation of the search strategy.

**Results:**

We illustrate our approach by means of an example, that is, a search for literature on brachytherapy in patients with prostate cancer. For this purpose, a test set was generated, including a total of 38 references from 3 systematic reviews. The development set for the generation of the strategy included 25 references. After application of textual analytic procedures, a strategy was developed that included all references in the development set. To test the search strategy on an independent set of references, the remaining 13 references in the test set (the validation set) were used. The validation set was also completely identified.

**Discussion:**

Our conclusion is that an objectively derived approach similar to that used in search filter development is a feasible way to develop and validate reliable search strategies. Besides creating high-quality strategies, the widespread application of this approach will result in a substantial increase in the transparency of the development process of search strategies.

## Background

Over the past few years, information retrieval has become more and more professionalized [1], and information specialists are considered full members of a research team conducting systematic reviews. Trial search coordinators in Cochrane Collaboration review groups are a good example of this development. They manage the search process in its entirety, from designing the search strategy to conducting and documenting the actual search and managing the references [2]. Information specialists also develop search filters that enable the efficient searching of bibliographic databases for specific methodological and subject-specific research questions [3-6].

Research groups preparing systematic reviews and clinical practice guidelines have been a main driving force in the development of search strategies, but they face challenges in terms of transparency and available resources. Various researchers and organizations have called for transparency in the documentation of search strategies in health technology assessment (HTA) reports and systematic reviews (SRs) [2,7,8], and new instruments have been developed for peer review of search strategies [9,10]. As SRs and HTA reports may inform health policy decisions and have far-reaching consequences, high demands on the transparency and validity of search strategies must be made in their development process. Time constraints play a decisive role in the development of search strategies, as information specialists often have no specific expert knowledge on many of the topics under investigation. This means that they must become acquainted with different topics within a short period of time, yet still be able to develop valid strategies. The traditional way to develop search strategies is to adopt a conceptual, that is, a concept-based, subjective approach. In our experience, however, this approach relies heavily on the information specialist's knowledge of the topic under investigation.

### Conceptual approach

The conceptual approach is recommended by the pertinent literature on the development of high-quality search strategies [2,11,12]. The key feature of this approach is the expertise of the searcher, that is, her or his knowledge of the database structure, the thesaurus and the research topic, as well as the clinicians' subject knowledge [6]. This means, for example, that when the search aims to retrieve literature on "rheumatoid arthritis," appropriate synonyms and related terms for the text word part of the strategy need to be identified. Different sources can help identify synonyms and related terms, for example, in medical dictionaries such as MedlinePlus or the entry terms of the MeSH (that is, medical subject heading) database. A similar procedure is used to identify controlled vocabulary. However, it remains unclear how to decide which terms to include in the search strategy. Furthermore, it is difficult, and might even be impossible, to tell when the strategy is completed. Several synonyms and related terms are conceivable in the above-described example, such as "juvenile rheumatoid arthritis," "Caplan syndrome," "Felty syndrome," "rheumatoid nodule," "Sjögren syndrome," "ankylosing spondylitis," "Still disease," "sicca syndrome," "Bechterew disease" and so on. The strategy becomes increasingly extensive but also more prone to error because more search queries are used, increasing the risk of spelling errors, logical operator errors, line number errors, truncation errors and so on. Another disadvantage of this approach is that the lack of criteria for selecting terms can lead to lengthy and often unproductive discussions among the research team.

#### Disadvantages of the conceptual approach

The following are disadvantages of the conceptual approach to search strategies: (1) It is difficult to determine when the search strategy is "complete"; (2) numerous search queries make the strategy more extensive but prone to error; (3) the conceptual approach is suited only for the development of a strategy, not for its validation; (4) if the retrieval rate is high, subsequent restriction of the search is required; and (5) it is time-consuming. A more objective way to generate and validate a search strategy for those parts of the search that are not covered by validated filters (for example, health condition, intervention) could help solve these difficulties.

### Learning from search filter development

In general, search filters are developed to search bibliographic databases efficiently, that is, to increase the number of relevant studies gathered while minimizing the number of irrelevant studies [13,14]. Search filters "are typically created by identifying and combining search terms to retrieve records with a common feature" [14] (p. 356). Attempts have been made to create different levels of strategies to cater to different users and their differing information needs [6]. The filters can be derived subjectively (expert-informed), objectively (research-based) or a combination of the two, that is, the search filter is derived subjectively but validated against a gold standard [6,14]. Information specialists use textual analysis software on a set of relevant references to identify representative terms in this set [3,6,15,16]. These empirically derived filters are then tested against a set of relevant and irrelevant records derived from a hand-search of SRs. There is general agreement that, whenever possible, objectively derived filters should be used.

Bak *et al. *[17] referred to Egger *et al. *[18] and stated that subjectively derived filters "draw their legitimacy from the expert knowledge ... and are therefore susceptible to the same criticisms as other reports of expert opinion" and that "as in standard biomedical evidence hierarchies, unvalidated filters based on expert opinion can be considered methodologically weak" [17] (p. 212).

#### Advantages of objectively derived search filters

The advantages of objectively derived search filters are that the design methods are clearly described and reproducible, empirically derived filters are developed on the basis of a set of relevant references and metrics (for example, sensitivity and precision) are applied to compare different filters.

To date, the development and testing processes used in filter development have not been applied by information specialists in the routine development of searches within the framework of SRs or HTA reports. Although some elements of the search strategy can be based on well-established search filters for certain research methods (for example, filters for randomized controlled trials (RCTs)), the content part (for example, health condition or intervention) of a search strategy is not usually tested, but some exceptions exist [19-22]. However, the advantages of filter development and the disadvantages of the traditional conceptual approach also apply to the routine process of search strategy development. The approach described below is an attempt to transfer the methods of developing and validating filters to those of search strategies.

### Objectives

On the basis of the example of brachytherapy for patients with prostate cancer, our aim in this paper is to describe the empirically guided development process for search strategies conducted by the German Institute for Quality and Efficiency in Health Care (Institut für Qualität und Wirtschaftlichkeit im Gesundheitswesen, or "IQWiG"). The paper is targeted mainly toward information specialists but may also provide useful information for other researchers with a specific interest in the development and validation of search strategies.

## Methods

### Implementation at IQWiG

HTA agencies and other institutions that regularly conduct SRs require robust and reliable search strategies. In practice, IQWiG uses the described method for various areas and study designs, for example, for clinical and health economic topics as well as for RCTs and observational studies.

Ideally, the quality of developed search strategies should be as high as that of methodological or topic-specific filters. IQWiG therefore applies a predefined approach to the development and validation of search strategies for SRs, which is outlined in its General Methods paper (version 4.0) [23]. This approach is used for all elements of a search strategy that cannot be based on a tested search filter and usually refers to the content part of the search strategy (health conditions and interventions). The process of the development and validation of search strategies for SRs consists of four steps: (1) generation of a test set, (2) development of the search strategy (objectively derived approach), (3) validation of the search strategy and (4) standardized documentation.

### Generation of a test set

To be able to develop and test a search strategy, a test set of relevant references is derived from SRs. For each HTA report, the information specialist conducts a preliminary search of the Cochrane Library (Cochrane Database of Systematic Reviews, Database of Abstracts of Reviews of Effects, and the Health Technology Assessment Database) to identify previous SRs in the area of interest. Because the Cochrane Collaboration specifies strict methodological standards for the preparation of SRs, it is a particularly trustworthy source for identifying this type of publication. When SRs on a similar research question are available and the search process, as well as the documented search strategy, is considered to be comprehensive, references included in the SRs are extracted to build the test set.

If SRs are not available, a precise strategy is developed and relevant articles are screened and selected by the review authors. For PubMed, the filter "Therapy/Narrow[filter]," which is accessed via the PubMed interface [24], is used for the precise search. In EMBASE, the precise filter "high specificity strategies" developed by McMaster University's Health Information Research Unit [25] can be accessed via Ovid.

The references identified in the SRs or the precise search are considered to be a "quasi-gold standard" [26]. The references identified are split randomly, using two-thirds for the development (development set) and one-third for the validation (validation set) of the search strategy.

### Development of the search strategy

After building the development set from the test set and importing the references into Endnote, a term frequency analysis is conducted using the Text Mining Package [27] of the R statistical software package [28]. On the basis of information derived from the titles and abstracts of the downloaded references, terms are ranked by frequency. Terms that are present in at least 20% of the references in the development set are selected for further examination. However, this ranking does not necessarily differentiate terms that are relevant to the research question from irrelevant terms in the target database. Therefore, a so-called population set consisting of a random sample of references is downloaded from the target database (for example, MEDLINE). This population set represents all references from the reference database and is compared to the development set. The most overrepresented terms related to the research question are used to develop the text word part of the search strategy. "Overrepresented" refers to the most frequent terms in the development set with a low sensitivity of 2% or less among the references in the population set [29]. The aim of this process is to identify those terms that are sensitive to the target references, but not to all references in the database.

Because of technical constraints, a simplified approach is adopted to identify controlled vocabulary. Terms are selected on the basis of their frequency in the development set and their relevance to the research question. For this purpose, tools such as PubMed PubReMiner, a free web service for searches in MEDLINE [30], or Endnote^®^, a reference management software are used. In PubReMiner subheadings should be used with caution: Because controlled vocabulary is listed individually as soon as different subheadings are used, they need to be summarized first. Only then is it possible to check how often controlled vocabulary actually appears in the articles.

The process described above identifies effective candidate terms: text terms and controlled vocabulary that might be suitable for inclusion in the search strategy. The candidate terms are allocated to three main sets of terms according to the definitions in the Cochrane Handbook [2]: (1) terms used to search for the health condition of interest, (2) terms entered to search for the intervention evaluated and (3) terms used to search for the types of study design to be included (validated search filters can usually be applied here [25,31-35]).

The next step in assembling these terms in the actual search is undertaken manually in an iterative trial-and-error approach. Because SRs usually aim to apply highly sensitive search strategies, the strategy should capture all references from the development set with sufficient precision to prevent the retrieval of too many irrelevant references. During the course of an IQWiG project, the search strategy may be adjusted in consultation with the project team: for example, if a high sensitivity results in an excessive number of hits, a more precise strategy may be required. The results of the textual analysis are drawn upon to enable an informed and transparent decision regarding a change in strategy.

### Validation of the search strategy

To confirm that the strategy developed works with a different set of references, the strategy is tested against a validation set. The validation set is also derived from SRs but contains different references than the development set. The strategy needs to be validated in the database for which the strategy was designed. The developed strategy is run in each database and compared to the validation set from that database using their accession numbers (for example, PMIDs in PubMed).

### Standardized internal documentation

To ensure transparency, each step of the process needs to be documented. This includes documentation of the preliminary or the precise search strategy, the SRs and relevant references used for the development of the search strategy, and frequency tables, including terms and controlled vocabulary. This comprehensive internal documentation can also be used to discuss search strategies and for quality assurance purposes.

## Results

To demonstrate the practical implementation of the described approach, in the following section we present the development of a search strategy for the content part of a search strategy applied to brachytherapy in patients with prostate cancer.

### Generation of a test set

**Figure 1:**
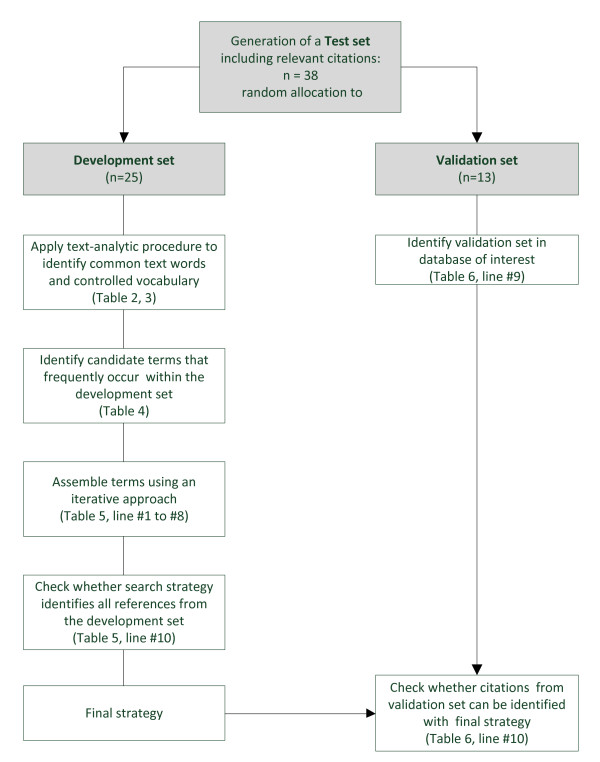
**Flowchart of an objectively derived approach using the example 'Brachytherapy in patients with prostate cancer'**.

We performed a search for SRs in the Cochrane Library (Table 1). Three SRs on brachytherapy in patients with prostate cancer were eligible publications, from which 38 relevant references were extracted for the generation of the test set. After random separation of the test set, 25 references were available for the development set and 13 were available for the validation of the strategy (see Figure 1).

**Table 1:** Cochrane Library search strategy^a^

ID	Search	Total hits
#1	(brachytherapy AND prostate):ti,ab,kw	124
#2	(#1) Cochrane Reviews (*n *= 1) Other reviews (*n *= 6) Technology assessments (*n *= 19)	26

^a ^Wiley search date 10 May 2011.

### Development of the search strategy

The analysis of text words of the 25 references from the development set, using the Text Mining Package in R, resulted in the generation of the list of frequencies in the development and population sets presented in Table 2. For example, the term "brachytherapy" was identified 19 times. As the development set included 25 references, this resulted in a sensitivity of 76%. A similar approach was chosen in the analysis of controlled vocabulary (listing them only according to frequency; see Table 3). For example, the term "brachytherapy" appeared a total of 20 times in the 25 references. A textual analysis was dispensed with for the study type, as validated study filters were available.

**Table 2:** Text words after analysis with the Text Mining Package in R (extract)

Terms	Frequency development set	Frequency population set	Sensitivity development set	Sensitivity population set
Patients	25	1,419	1.0000	0.1976
Results	25	1,425	1.0000	0.1985
Cancer	24	361	0.9600	0.0503
Methods	24	335	0.9600	0.0467
Prostate	24	49	0.9600	0.0068
Treatment	21	941	0.8400	0.1311
External	20	86	0.8000	0.0120
Therapy	20	342	0.8000	0.0476
Treated	20	371	0.8000	0.0517
Beam	19	32	0.7600	0.0045
Brachytherapy	19	4	0.7600	0.0006
Compared	17	972	0.6800	0.1354
Conclusions	17	41	0.6800	0.0057
Follow	16	49	0.6400	0.0068
Prostatectomy	16	11	0.6400	0.0015
Radical	16	52	0.6400	0.0072
Respectively	16	72	0.6400	0.0100
Risk	16	584	0.6400	0.0813
Specific	16	488	0.6400	0.0680
Localized	15	66	0.6000	0.0092
Months	15	259	0.6000	0.0361
Purpose	15	192	0.6000	0.0267
Radiation	15	68	0.6000	0.0095
Analysis	14	853	0.5600	0.1188
Radiotherapy	14	30	0.5600	0.0042
Rates	14	260	0.5600	0.0362
Score	14	115	0.5600	0.0160
Significantly	14	873	0.5600	0.1216
Biochemical	13	87	0.5200	0.0121
Gleason	13	4	0.5200	0.0006
Materials	13	162	0.5200	0.0226
Antigen	12	76	0.4800	0.0106
Time	12	576	0.4800	0.0802
Using	12	1,680	0.4800	0.2340

**Table 3:** Controlled vocabulary after frequency analysis with PubMed PubReMiner (extract)

Terms	Frequency
Humans	23
Male	23
Prostatic neoplasms	23
Aged	20
Brachytherapy	20
Middle aged	17
Prostate-specific antigen	12
Prostatectomy	12
Adenocarcinoma	9
Follow-up studies	8
Retrospective studies	8
Aged, 80 and over	8
Disease-free survival	7
Radiotherapy dosage	6
Adult	6
Neoplasm staging	5
Proportional hazards models	5
Quality of Life	4
Prospective studies	4
Radiotherapy, conformal	4
Incidence	3
SEER program	3
Iodine radioisotopes	3
Risk factors	3
Combined modality therapy	3
Risk assessment	3
Survival rate	3
Treatment outcome	3
Multivariate analysis	3
Questionnaires	3

### Generation of the candidate terms

Taking the relevance of the topic into account, candidate terms were extracted from both lists and displayed in a new list. These candidate terms were allocated to one of three categories: health condition, intervention and "questionable terms" (terms for which it was unclear whether they should be considered in the strategy as well as terms that required further assessment) (see Table 4 and Additional material). In this context, it should be noted that "questionable terms" may also include terms that do not directly represent the intervention or health condition of interest. In our example, "Gleason" is a score for histologic grading. Such a term needs to be clarified *a priori*. The inclusion in the search strategy would be considered only if the specific terms did not identify the references from the test set in the categories "health condition" and "intervention."

**Table 4:** Candidate terms (sorted)

Category	Candidate terms
Health condition	
Prostatic neoplasms	Controlled vocabulary
Prostate	Text terms
Adenocarcinoma	Text terms
Cancer	Text terms
Intervention	
Brachytherapy	Controlled vocabulary
Brachytherapy	Text terms
Seed	Text terms
Permanent	Text terms
Implantation	Text terms
Questionable terms	
Iodine radioisotopes	Controlled vocabulary
Prostate-specific antigen	Controlled vocabulary
Localized	Text terms
Gleason	Text terms
PSA	Text terms

The development of the search strategy was based on a trial-and-error approach whereby the candidate terms identified were entered into the bibliographic database with the corresponding syntax and we tested whether references from the development set could be detected (see line 8 of the search strategy in Table 5). In the example presented, the 25 hits of the development set were identified with the search strategy, meaning that sensitivity reached 100% (line 10 of the search strategy in Table 5). This means that there was no need to use questionable terms and that the search strategy could then be tested by means of the validation set.

**Table 5:** MEDLINE search strategy (prefinal)^a^

Number	Searches	Results
1	Prostatic Neoplasms/	78,913
2	(prostat* and (cancer or adenocarcinoma)).ab,ti.	73,416
3	or/1-2 [Health condition]	95,729
4	Brachytherapy/	13,758
5	Brachytherapy.ab,ti.	9,822
6	((seed* or permanent*) and implant*).ab,ti.	10,719
7	or/4-6 [Intervention]	25,031
8	and/3,7	3,347
9	("18374503" or "11104883" or "10924979" or "15541117" or "18963536" or "15590163" or "14665356" or "9749478" or "11490252" or "18207665" or "18325680" or "19455340" or "2009027580" or "10792092" or "14697417" or "18374892" or "18801517" or "20427255" or "19570619" or "15066293" or "15737905" or "20378156" or "19670452" or "10080594" or "18538495").ui. [development set]	25
10	8 and 9	25

^a^Ovid search date 19 September 2011. Ovid MEDLINE in-process and other nonindexed references, Ovid MEDLINE daily and Ovid MEDLINE, 1950 to present.

### Validation of the search strategy

The last step comprised the validation of the developed search strategy. For this purpose, the 13 references previously identified from the validation set were used. All references from the validation set could be identified (see line 10 of the search strategy in Table 6).

**Table 6:** MEDLINE validation of a search strategy with a validation set^a^

Number	Searches	Results
1	Prostatic Neoplasms/	78,913
2	(prostat* and (cancer or adenocarcinoma)).ab,ti.	73,416
3	or/1-2 [Health condition]	95,729
4	Brachytherapy/	13,758
5	Brachytherapy.ab,ti.	9,822
6	((seed* or permanent*) and implant*).ab,ti.	10,719
7	or/4-6 [Intervention]	25,031
8	and/3,7	3,347
9	("15476513" or "17293235" or "17570425" or "20399462" or "19571899" or "11597800" or "20303100" or "19376564" or "20231039" or "12084197" or "19945997" or "10758314" or "14581420").ui. [validation set]	13
10	8 and 9	13

^a^Ovid search date 19 September 2011. #1 through #8, search strategy developed; #9, search string with accession numbers of the validation set; and #10, validation of the strategy with the validation set. Ovid MEDLINE in-process and other nonindexed references, Ovid MEDLINE daily and Ovid MEDLINE 1950 to present.

### Standardized internal documentation

During the development and validation process, the following documents were stored for later quality control: the three SRs from which the test set was generated [36-38]; the frequency tables, that is, the results of the textual analysis (Tables 2 and 3); the extraction of the candidate terms (Table 4); the prefinal search strategy (Table 5); and the validation results (Table 6).

## Discussion

### 

Search strategies for SRs and HTA reports can be developed and validated using an objectively derived approach which includes elements such as the use of a test set (quasi-gold standard) as a reference standard. This type of approach is already being widely applied in the development of filters and is the current standard applied by IQWiG.

Our screening of other HTA agency websites indicates that they rarely describe their approach to the development and validation of search strategies. One exception is the Danish Centre for Health Technology Assessment, which in its manual outlines a pragmatic approach to the validation of search strategies [12]. The Swedish Council on Technology Assessment in Health Care mentions "inverse searching," which "is done by taking articles that are already known to be relevant to the assessment and locating their references (including their indexing terms) in the database. By inspecting the indexing terms of those references, searchers can determine how relevant articles are indexed, and can use these indexing terms to retrieve more relevant references" [39]. Patrick *et al. *stated that search strategies of meta-analyses should report evidence of the effectiveness of their retrieval strategies, for example, by the use of a previously tested search strategy [40]. Although this might be a useful approach, to date it remains rare in search development. Existing instruments such as the recently published peer review instrument PRESS by Sampson *et al. *[10], which is designed to review subjectively developed search strategies, contain no performance-oriented assessment criteria that can be reported on the basis of the objective approach described above. Although the examples named above lack a systematic and comprehensive approach, they show that the demand for an objective approach to the development and validation of search strategies is increasingly being recognized.

The success of empirically developed search filters is judged by the generalizability of the gold standard. So far, hand-searching has been considered the method of choice. This approach is rather costly, and hand-searches are therefore often performed in only a small number of journals and volumes. An alternative approach was described and applied by Sampson *et al. *[26], who also extracted relevant references from SRs and noted that "recall is only as good as the sum of the individual searches" [26]. To counter this limitation, as a rule IQWiG performs a quality control of the search process of the SRs to be included. When the search process employed is considered to be comprehensive (multiple sources and traditional techniques to identify relevant articles), the references found seem to be more representative (more journals and volumes) of the targeted pool of relevant references. This statement is supported by the findings of Simon *et al. *[41] who compared both ways of developing a gold standard and concluded that with increasing numbers of relevant references, differences between hand-searching and SRs could be neglected. If only minor differences between hand-search-generated gold standards or SR-based quasi-gold standards were noted, this might offer the opportunity to apply methods usually used in search filter development to the routine development of search strategies.

#### Challenges for the future

Objectively derived and validated search strategies are an essential contribution to the development of high-quality search strategies. Some questions remain unanswered, however, and need to be addressed in future research. For instance, it is unclear how to handle situations where SRs are lacking or fail to fully cover the topic of interest. One approach could be to combine the concepts of interest from different SRs. For example, if the use of positron emission tomography (PET) in patients with gliomas is to be investigated, it might be appropriate to generate relevant references for this intervention, for example, from an SR on PET in patients with lymphoma, head and neck cancer and so on, and also to consider another SR that, for example, investigates the use of chemotherapy in patients with gliomas. This approach would ensure that a sufficient number of references would be retrieved to develop and validate single parts of the search strategy.

Another critical issue in the development and validation process is to determine the optimal number of references. So far, our experience shows that the suggested approach to develop the search strategy can even be used with a small sample of references. However, future research should explore sample size requirements for the development and validation process.

#### Statistical methods to build the strategy

At IQWiG, we currently still use an iterative and essentially subjective approach to building the actual search strategy, which could be viewed as a limitation. Statistical approaches such as logistic regression or factor analysis [16] might be ways to find a more objective approach to performing this step. Further research is needed to determine whether these techniques produce competitive search strategies within an acceptable time frame.

### Summary of strengths and weaknesses of the objectified approach

#### Strengths

The strengths of the objective approach are that it is transparent, it makes informed decisions possible with regard to the inclusion of terms and it allows information specialists to work more independently.

#### Weaknesses

The weaknesses of the objective approach are that, depending on the topic, only a few relevant articles may be available for textual analysis; it is a "one-shot" search strategy, because before applying the strategy again it has to be tested once more; and methodological challenges remain.

## Conclusion

Conceptual approaches have traditionally been used in the development of search strategies, but they lack objectivity and validity. An objectively derived approach similar to that used in search filter development is a feasible way to develop and validate reliable search strategies. Besides creating high-quality strategies, the widespread application of this approach would result in a substantial increase in the transparency of the development process. To promote its implementation, the use of an objective approach could be added to checklists as an item for the quality assurance of search strategies. Further research is required on the development of statistical methods for building the actual search strategy.

## Competing interests

The authors declare that they have no competing interests.

## Authors' contributions

EH coordinated the planning of the study, data collection, search strategy development, data analysis, interpretation of the results, drafting and revision of the manuscript. SW participated in the review of the literature, interpretation of the results and revision of the manuscript. MS and TK participated in the interpretation of the results and the revision of the manuscript. All authors read and approved the final manuscript.

## Supplementary Material

Additional file 1 **Candidate terms: text terms**. Click here for file

Additional file 2 **Candidate terms: controlled vocabulary**. Click here for file
