# Supplementary material for: Routine development of objectively derived search strategies
Source: Syst Rev. 2012 Feb 29;1:19. doi: 10.1186/2046-4053-1-19 (PMC3351720; doi:10.1186/2046-4053-1-19)
Supplement: Additional file 1 — Candidate terms: text terms. [file 2046-4053-1-19-S1.PDF]

Term	Frequency development set	Frequency population set	Sensitivity development set	Sensitivity population set
brachytherapy	19	4	0,7600	0,0006
gleason	13	4	0,5200	0,0006
psa	11	8	0,4400	0,0011
urological	5	8	0,2000	0,0011
prostatectomy	16	11	0,6400	0,0015
oncology	9	13	0,3600	0,0018
androgen	6	14	0,2400	0,0019
seed	5	15	0,2000	0,0021
permanent	8	19	0,3200	0,0026
deprivation	6	19	0,2400	0,0026
adenocarcinoma	8	22	0,3200	0,0031
modality	7	22	0,2800	0,0031
conclusion	8	27	0,3200	0,0038
relapse	6	29	0,2400	0,0040
radiotherapy	14	30	0,5600	0,0042
beam	19	32	0,7600	0,0045
worse	9	33	0,3600	0,0046
implantation	6	34	0,2400	0,0047
conclusions	17	41	0,6800	0,0057
cox	6	41	0,2400	0,0057
definition	6	41	0,2400	0,0057
consensus	5	45	0,2000	0,0063
prostate	24	49	0,9600	0,0068
follow	16	49	0,6400	0,0068
radical	16	52	0,6400	0,0072
society	5	54	0,2000	0,0075
grade	8	60	0,3200	0,0084
symptom	5	60	0,2000	0,0084
localized	15	66	0,6000	0,0092
biopsy	5	66	0,2000	0,0092
radiation	15	68	0,6000	0,0095
intermediate	7	68	0,2800	0,0095
respectively	16	72	0,6400	0,0100
american	9	75	0,3600	0,0104
antigen	12	76	0,4800	0,0106
urinary	7	77	0,2800	0,0107
database	6	78	0,2400	0,0109
toxicity	5	81	0,2000	0,0113
external	20	86	0,8000	0,0120
biochemical	13	87	0,5200	0,0121
late	7	97	0,2800	0,0135
cohort	5	108	0,2000	0,0150
multivariate	5	110	0,2000	0,0153
clinically	8	113	0,3200	0,0157
scores	8	113	0,3200	0,0157
score	14	115	0,5600	0,0160
stage	11	121	0,4400	0,0169
free	9	132	0,3600	0,0184
failure	11	138	0,4400	0,0192
retrospective	5	147	0,2000	0,0205
median	10	149	0,4000	0,0208
defined	8	154	0,3200	0,0214
dose	6	158	0,2400	0,0220
materials	13	162	0,5200	0,0226
compare	7	163	0,2800	0,0227
outcomes	8	175	0,3200	0,0244
incidence	6	175	0,2400	0,0244
life	7	178	0,2800	0,0248
regression	6	188	0,2400	0,0262
purpose	15	192	0,6000	0,0267
initial	6	201	0,2400	0,0280
comparison	8	203	0,3200	0,0283
survival	9	208	0,3600	0,0290
versus	7	209	0,2800	0,0291
received	6	218	0,2400	0,0304
independent	5	225	0,2000	0,0313
surgery	6	227	0,2400	0,0316
overall	9	241	0,3600	0,0336
improved	7	243	0,2800	0,0338
relative	6	249	0,2400	0,0347
therapeutic	6	252	0,2400	0,0351

Health condition
prostate
adenocarcinoma
cancer

Intervention
brachytherapy
seed
permanent
implantation

Questionable terms
localized
gleason
psa

quality	7	254	0,2800	0,0354
months	15	259	0,6000	0,0361
rates	14	260	0,5600	0,0362
population	5	261	0,2000	0,0364
outcome	8	274	0,3200	0,0382
difference	5	296	0,2000	0,0412
association	7	303	0,2800	0,0422
acute	5	316	0,2000	0,0440
methods	24	335	0,9600	0,0467
therapy	20	342	0,8000	0,0476
single	5	352	0,2000	0,0490
cancer	24	361	0,9600	0,0503
treated	20	371	0,8000	0,0517
included	6	374	0,2400	0,0521
primary	6	378	0,2400	0,0526
differences	6	419	0,2400	0,0584
level	6	432	0,2400	0,0602
rate	10	433	0,4000	0,0603
following	7	434	0,2800	0,0604
age	8	452	0,3200	0,0630
similar	9	456	0,3600	0,0635
determine	5	458	0,2000	0,0638
lower	7	469	0,2800	0,0653
specific	16	488	0,6400	0,0680
low	9	489	0,3600	0,0681
patient	6	537	0,2400	0,0748
total	5	565	0,2000	0,0787
control	7	574	0,2800	0,0799
time	12	576	0,4800	0,0802
risk	16	584	0,6400	0,0813
disease	7	600	0,2800	0,0836
group	5	615	0,2000	0,0857
based	6	666	0,2400	0,0928
analysis	14	853	0,5600	0,1188
significantly	14	873	0,5600	0,1216
clinical	6	925	0,2400	0,1288
treatment	21	941	0,8400	0,1311
significant	10	946	0,4000	0,1318
compared	17	972	0,6800	0,1354
associated	5	1002	0,2000	0,1396
data	9	1022	0,3600	0,1423
patients	25	1419	1,0000	0,1976
results	25	1425	1,0000	0,1985
between	5	1674	0,2000	0,2331
using	12	1680	0,4800	0,2340
study	11	1864	0,4400	0,2596
this	5	3282	0,2000	0,4571
and	17	6126	0,6800	0,8532
the	22	6156	0,8800	0,8574
