# Supplementary material for: Routine development of objectively derived search strategies
Source: Syst Rev. 2012 Feb 29;1:19. doi: 10.1186/2046-4053-1-19 (PMC3351720; doi:10.1186/2046-4053-1-19)
Supplement: Additional file 2 — Candidate terms: controlled vocabulary. [file 2046-4053-1-19-S2.PDF]

Term	Frequency
Humans	23
Male	23
Prostatic Neoplasms	23
Aged	20
Brachytherapy	20
Middle Aged	17
Prostate-Specific Antigen	12
Prostatectomy	12
Adenocarcinoma	9
Follow-Up Studies	8
Retrospective Studies	8
Aged, 80 and over	8
Disease-Free Survival	7
Radiotherapy Dosage	6
Adult	6
Neoplasm Staging	5
Proportional Hazards Models	5
Quality of Life	4
Prospective Studies	4
Radiotherapy, Conformal	4
Incidence	3
SEER Program	3
Iodine Radioisotopes	3
Risk Factors	3
Combined Modality Therapy	3
Risk Assessment	3
Survival Rate	3
Treatment Outcome	3
Multivariate Analysis	3
Questionnaires	3
Analysis of Variance	2
Neoplasms, Radiation-Induced	2
Risk	2
Palladium	2
Radioisotopes	2
Erectile Dysfunction	2
Prostate	2
Survival Analysis	2
Androgen Antagonists	2
Cross-Sectional Studies	2
Radiation Injuries	2
Radiotherapy, Intensity-Modulated	2
Radiotherapy	2
Cohort Studies	2
Prognosis	2
Age Factors	1
Chi-Square Distribution	1
Neoplasms, Second Primary	1
DNA Mutational Analysis	1
Urinary Incontinence	1

Health condition

Prostatic Neoplasms

Intervention

Brachytherapy

Questionable terms

Iodine Radioisotopes
Prostate-Specific Antigen

Biopsy	1
Regression Analysis	1
Computer Systems	1
Intraoperative Care	1
Life Tables	1
Magnetic Resonance Imaging	1
Neoplasm Invasiveness	1
Tumor Markers, Biological	1
Antineoplastic Agents, Hormonal	1
Chemotherapy, Adjuvant	1
Data Collection	1
Acute Disease	1
Chronic Disease	1
Gastrointestinal Tract	1
Urogenital System	1
Health Status	1
Spain	1
Postoperative Complications	1
Colon	1
Colonic Diseases	1
Diarrhea	1
Gastrointestinal Hemorrhage	1
Proctitis	1
Rectum	1
Age Distribution	1
Confidence Intervals	1
Rectal Neoplasms	1
Registries	1
Urinary Bladder Neoplasms	1
Leukemia, Myeloid, Acute	1
Population Surveillance	1
Matched-Pair Analysis	1
Neoadjuvant Therapy	1
Reference Values	1
Time Factors	1
Animals	1
Kidney Diseases	1
Mice	1
Morbidity	1
Urination Disorders	1
Ohio	1
Prevalence	1
